# An Ensemble Spatiotemporal Model for Predicting PM_2.5_ Concentrations

**DOI:** 10.3390/ijerph14050549

**Published:** 2017-05-22

**Authors:** Lianfa Li, Jiehao Zhang, Wenyang Qiu, Jinfeng Wang, Ying Fang

**Affiliations:** 1 State Key Laboratory of Resources and Environmental Information Systems, Institute of Geographic Sciences and Natural Resources Research, Chinese Academy of Sciences, No A11, Datun Road, Beijing 100101, China; zhangjh@lreis.ac.cn (J.Z.); qiuwy@lreis.ac.cn (W.Q.); wangjf@lreis.ac.cn (J.W.); fangy.16s@igsnrr.ac.cn (Y.F.); 2 University of Chinese Academy of Sciences, Beijing 100049, China

**Keywords:** PM_2.5_, PM_10_ predictor, exposure estimation, kriging, ensemble model

## Abstract

Although fine particulate matter with a diameter of <2.5 μm (PM_2.5_) has a greater negative impact on human health than particulate matter with a diameter of <10 μm (PM_10_), measurements of PM_2.5_ have only recently been performed, and the spatial coverage of these measurements is limited. Comprehensively assessing PM_2.5_ pollution levels and the cumulative health effects is difficult because PM_2.5_ monitoring data for prior time periods and certain regions are not available. In this paper, we propose a promising approach for robustly predicting PM_2.5_ concentrations. In our approach, a generalized additive model is first used to quantify the non-linear associations between predictors and PM_2.5_, the bagging method is used to sample the dataset and train different models to reduce the bias in prediction, and the variogram for the daily residuals of the ensemble predictions is then simulated to improve our predictions. Shandong Province, China, is the study region, and data from 96 monitoring stations were included. To train and validate the models, we used PM_2.5_ measurement data from 2014 with other predictors, including PM_10_ data, meteorological parameters, remote sensing data, and land-use data. The validation results revealed that the R^2^ value was improved and reached 0.89 when PM_10_ was used as a predictor and a kriging interpolation was performed for the residuals. However, when PM_10_ was not used as a predictor, our method still achieved a CV R^2^ value of up to 0.86. The ensemble of spatial characteristics of relevant factors explained approximately 32% of the variance and improved the PM_2.5_ predictions. The spatiotemporal modeling approach to estimating PM_2.5_ concentrations presented in this paper has important implications for assessing PM_2.5_ exposure and its cumulative health effects.

## 1. Introduction

Studies show that air pollution has negative health impacts on humans [1], and this issue has received considerable attention in recent years [2,3]. The air pollutants that are most dangerous to humans include sulfur dioxide, nitrogen dioxide, ozone, and particulate matter with an aerodynamic diameter of less than 2.5 μm (PM_2.5_) [4]. Because of its small size, particulate matter can adhere to the deep respiratory tract and affect blood circulation by penetrating lung cells [5,6]. Several studies have shown that particulate matter increases the risk of developing airway obstructive disease, chronic bronchitis [7], asthma (in children) [8,9,10], lung cancer [11], and various other cardiovascular diseases [12,13,14]. Thus, when assessing PM_2.5_ pollution and the cumulative health effects, an accurate method of estimating PM_2.5_ exposure at fine spatial and temporal resolutions is essential, even when actual measurements are unavailable. However, the global monitoring of PM_2.5_ remains in a nascent phase and is characterized by limited spatial coverage, as observed in China [15,16].

Due to missing early PM_2.5_ monitoring data, and the insufficient spatiotemporal coverage of existing PM_2.5_ monitoring data, the approaches to estimating PM_2.5_ exposure have not been developed fully until recently. Early practitioners [17,18] used simple methods to predict PM_2.5_ at time slices when measurements of co-located pollutants (PM_10_ or total suspended particulates (TSPs)) were available. These approaches involved the use of long-term ratios of PM_2.5_ to the co-located pollutants, which introduced considerable limitations because of the lack of available data for these pollutants.

Advanced approaches have recently been applied for PM_2.5_ exposure estimations, including the land-use regression (LUR) [19] and kriging methods [20]. Further, the increased availability of satellite data has led to strategies that employ normalized difference vegetation index (NDVI), surface temperature, and aerosol optical thickness (AOT) data in combination with other non-satellite variables, such as meteorological parameters, traffic indices and elevation levels [21]. Of these recent approaches, linear regressions have been applied extensively, although non-linear approaches have also been used [22]. Kriging is often used separately from LUR because of the model’s complexities in combining other covariates. In addition, several spatiotemporal models have been developed for the estimation of PM_2.5_ concentrations at high spatiotemporal resolutions. Kloog et al. [23] proposed a number of spatiotemporal models using satellite-derived AOT and they obtained an out-of-sample average R^2^ of 0.81. Xie et al. [24] and Zheng et al. [25] employed satellite-derived AOT data for China to predict the daily PM_2.5_ levels and achieved a R^2^ of roughly 0.80. However, the two AOT-based approaches measure pollution at a coarse spatial resolution (three kilometers) and cannot reliably estimate the within-community variability of PM_2.5_ at higher spatial resolutions. For the kriging methods, measurement data are usually used to train variogram models to perform PM_2.5_ concentration predictions without considering other covariates [20] that might limit the model’s predictive power.

Due to the limited temporal and spatial coverage of PM_2.5_ monitoring data, as well as the limitation of the previous estimation methods, we propose an ensemble spatiotemporal modeling approach to be an improvement in estimating PM_2.5_ concentrations. Compared with the previous methods, our approach integrates non-linear associations, ensemble learning, and residual kriging methods to predict the within-community variability of PM_2.5_ with an improved accuracy. Our approach employed generalized additive models (GAM) to consider the variability of spatial and spatiotemporal predictors with non-linear effects to capture associations between predictors and PM_2.5_ [26]. Ensemble learning can be used to generate stable predictions with less extreme values based on multiple GAM models while also outputting an uncertainty indicator (standard deviation). A kriging interpolation of the daily residuals derived from ensemble learning predictions can be used to capture residual spatial patterns and considerably improve estimations even without use of PM_10_ or other co-pollutant predictor. We also examined the proposed approach for several scenarios of different combinations of predictors, and we demonstrated how our approach could achieve optimal accuracy for these different scenarios. Supplemental Materials Table S1 presents an annex table for technical terms used in this paper.

## 2. Materials and Methods

### 2.1. Study Domain

Shandong Province, China, was the study region, and it is located between latitude 34°25′ and 38°23′ N and longitude 114°35′ and 112°43′ E. In 2014, the province covered an area of 157,900 km^2^ and had a population of roughly 97.89 million. The study domain is a typical northern province of China that is heavily polluted by PM_2.5_. We have a complete monitoring data of PM_2.5_ and PM_10_ with a lot of covariates available. Thus, we chose this region as the study domain for our approach.

### 2.2. Monitoring Data

**Figure 1:**
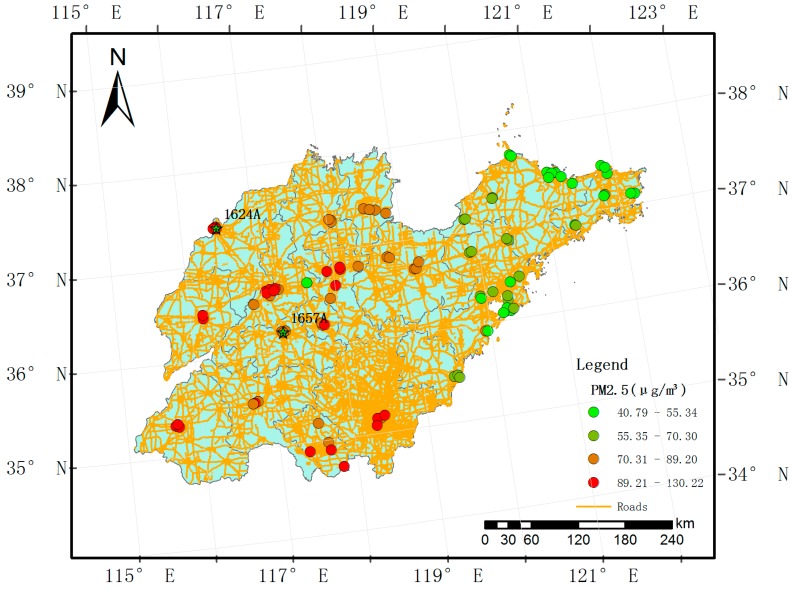
Study region (Shandong Province in China) with monitoring stations.

**Figure 2:**
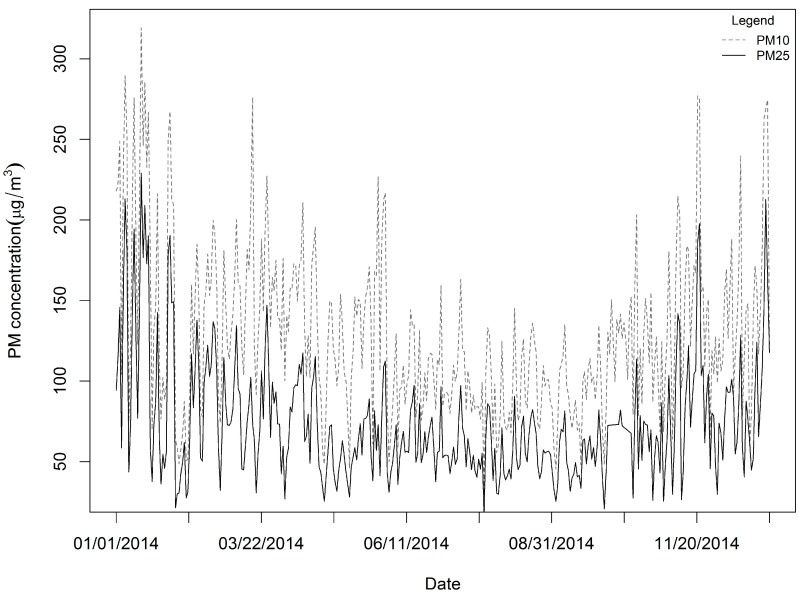
Daily averages of PM_2.5_ and PM_10_ over all the monitoring stations in 2014.

PM_2.5_ and PM_10_ concentration data (unit: μg/m^3^) were obtained from the Ministry of Environmental Protection of the People’s Republic of China (http://datacenter.mep.gov.cn/). The data were gathered at 96 national air quality monitoring stations located in 17 cities (Figure 1) from 1 January to 31 December 2014 (365 days). Because 15 days of data were missing, we imputed the omitted data using the singular value decomposition-based missing-value method [27] using the pcaMethods package (Max-Planck Institute for Molecular Plant Physiology, Golm, Germany), which is available through the R statistical software program (version 3.2.2). PM_10_ and PM_2.5_ concentrations were monitored hourly, and we calculated the daily average for each site using the 75% measurement standard (measurements covering 75% of a 24-h period were used to calculate the daily means). The daily mean concentration for 2014 is shown in Figure 2. In 2014, monitoring stations in the northwest region of Shandong Province presented lower average PM_2.5_ concentrations than those of the central and southern regions (Figure 1).

### 2.3. Predictors

We collected predictor data from multiple sources, including meteorological parameters, remote sensing derived variables, traffic indices, point emissions, land uses, and elevation levels. Meteorological parameters such as air temperature, precipitation, humidity, and wind speed are closely associated with concentrations of particulate matter, particularly PM_2.5_ [28,29,30,31,32]. Data from the Modern-Era Retrospective Analysis for Research and Applications were used to extract the meteorological parameters, including the surface air temperature (°C), effective surface specific humidity (kg/kg), bias-corrected total precipitation (kg/m^2^s), surface eastward wind speed (m/s), and surface northward wind speed levels (m/s). The Modern-Era Retrospective Analysis dataset is a NASA reanalysis dataset for the satellite era that integrates a variety of observational systems to produce a temporally and spatially consistent synthesis [33,34]. The spatial resolution of the meteorological parameters was 0.5° latitude by 0.6° longitude, and an hourly temporal resolution was used [34]. For the meteorological predictors, we calculated the average of the 24-h measurements for each day.

Transportation emissions constitute one source of air pollution emissions [26,35,36,37]. We extracted two index variables as predictors of transportation: the road lengths of the 10 km buffer zones around each monitoring station and the shortest distance from each station to the nearest road. We used the Open Street Map (http://www.openstreetmap.org/) as the road network for deriving traffic predictors.

In addition to traffic predictors, industrial plants, such as coal, oil-burning, chemical, smelting, power, paper, and mining plants, may constitute major sources of PM_2.5_ emissions [38]. To reflect the emission sources for PM_2.5_, we obtained location information on 326 plants in the study domain via Baidu map points of interest technologies (a major map provider in China). We measured the number of plants potentially releasing PM_2.5_ pollution within the optimal 10 km buffer of each monitoring station as well as the shortest distance between each station and its closest plant.

Land-use data for 2010 were obtained from the Chinese Academy of Sciences Data Center for Resources and Environmental Sciences [39]. The dataset consists of six first-class types (plow areas, forest land, grassland, water bodies, residential areas, and unutilized areas) and 25 second-class types with a 1 km resolution. We extracted the proportion of the forest land-use areas as an environmental factor and the proportion of construction land-use areas (referring to land used for factories and mines, oil fields, and stone pits) as an indirect emission factor within the optimal 10 km buffer (with the highest correlation to PM_2.5_) of the monitoring stations.

Aerosol optical thickness (AOT) data were extracted from satellite images [40]. More specifically, AOT data (spatial resolution: 1 km; temporal resolution: daily) derived from the Moderate Resolution Imaging Spectroradiometer MODIS Terra/Aqua satellites were used, and we observed a strong correlation between the satellite AOT data and PM_2.5_ levels in certain regions of China [41,42]. In addition, we extracted the monthly NDVI (normalized difference vegetation index) from the MODIS MODND1D dataset (spatial resolution: 500 m; temporal resolution: monthly) for each sampling location. The dataset was provided by the International Scientific and Technical Data Mirror Site Computer Network Information Center of the Chinese Academy of Sciences [43].

In addition to the predictors described above, we considered GDP and digital elevation model (DEM) values. GDP denotes the economic level of the study domain, and DEM values reflect the terrain characteristics that may influence pollution diffusion [44]. However, the data were not significantly correlated with PM_2.5_; therefore, the two variables were not considered in the models.

Variables were selected based on the following two steps. First, we calculated the variance inflation factor (VIF) to avoid multicollinearity [45]. Second, we used the backward-stepwise method to select variables for each combination by removing a variable once until the Akaike’s information criterion (AIC) [46] remained the same. Finally, we selected variable combinations with the lowest AIC values as optimal inputs.

### 2.4. Modeling Approach

This section describes our modeling process from the generation of the predictors of temporal basis trends (Section 2.4.1), non-linear additive modeling (Section 2.4.2), ensembling learning (Section 2.4.3), kriging of the residuals (Section 2.4.4), and validation and independent test (Section 2.4.5).

#### 2.4.1. Generation of the Predictors of Temporal Trends

**Figure 3:**
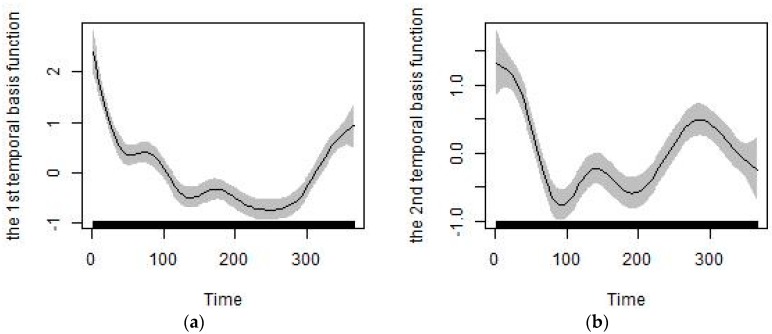
Temporal basis functions for Shandong Province of China: (**a**) The first temporal basis function; (**b**) The second temporal basis function.

Besides the meteorological, remote sensing, traffic, point emission, land-use and elevation predictors listed in Section 2.3, we also extracted the predictors of temporal trends from the temporally continuous monitoring PM_2.5_ data. Air pollutant levels, including PM_2.5_, follow a regular temporal trend of high values in winter and low values in summer [47]. Thus, we can use the temporal variables extracted to model seasonal trends for the study region. Of the two methods, empirical mode decomposition [48] and singular value decomposition (SVD) [49,50], we used the SVD method to obtain the temporal basis functions and capture dominant seasonal trends because of its better generalizability and missing data processing capabilities. Cubic smoothing splines were used to fit the temporal basis function, and the first and second temporal basis functions for the Shandong province of China are shown in Figure 3. The first and second temporal basis functions were used as predictors as they account for a majority of the seasonal variability of PM_2.5_ for the study region [51].

#### 2.4.2. Non-Linear Additive Modeling

The following equation illustrates our non-linear modeling framework:

(1) y(s,t)=μ(s,t)+ε(s,t),  y(s,t)~N(μ,σ)

(2) $$\mu \left(s,t\right)=f_{1}(t)+\;f_{2}\left(t\right)+\sum_{i=1}^{n}s\left(x_{i}\left(s,t\right)\right)+\sum_{k=1}^{m}s\left(p_{k}\left(s\right)\right)$$

In Equation (1), *s* and *t* represent the location (latitude and longitude) and time (day index), respectively, of the monitoring sample; y(s,t) represents the log-transformed concentration of measured or estimated PM_2.5_ (μg/m^3^), because the histograms of the PM_2.5_ and PM_10_ levels (Supplemental Materials Figure S1) showed a right-skewed distribution; thus, log transformation was necessary for normalization; and *μ*(*s*, *t*) represents the mean for y(s,t) with spatiotemporal residual *ε*(*s*, *t*) ~ N(0, *σ*^2^). In Equation (2), the mean of *μ*(*s*, *t*) are modeled based on temporal variables (the first and second temporal basis functions $f_{1}\left(t\right)$ and $f_{2}\left(t\right)$ and spatiotemporal variables (*x_i_*(*s*, *t*) including co-located PM_10_, meteorological parameters, AOT, and NDVI) and spatial variables (*p_k_*(*s*), including traffic indices and land-use predictors). We used generalized additive models (GAMs) to perform the non-parametric non-linear associations (*s*(*…*) refers to smooth functions), and we set the maximum degree of freedom to 10 to prevent overfitting in the non-linear model [49].

The “mgcv” package available through the R statistical software program (version 3.2.2) was used to model the non-linear additive modeling in Equation (2).

#### 2.4.3. Ensemble Learning

Section 2.4.1 presents the individual GAM models, which may be sensitive to new datasets when performing practical predictions. The ensemble method of machine learning [52] was used to enhance the predictions of the multiple GAM models included in Equations (1) and (2) and to avoid the potential over-fitting problem; meanwhile, standard deviation can be obtained from multiple predictions as an uncertainty indicator. Because of the strong performance of bootstrap aggregating (bagging) methods [53,54,55,56,57], we used bagging to conduct integrated learning for the individual models. In this method, input data are first sampled with replacements to generate multiple sets of input data of the same size as the original input data. Using different sample subsets, various regression models were trained. Cross-validations were used to determine the optimal number of models, and we ultimately obtained 1000 models to capture the dataset’s different variability levels based on the varied sample subsets. Each trained model was then used to make predictions for the remaining sample to train the model [57]. Because the original dataset included 35,040 data points, we obtained a re-sampled training dataset (63.2% of the original dataset) and remaining test data (36.8%) for independent validation [58]. The final integrated prediction was the mean weighted by the model’s performance (measured by R^2^):

(3) $$\mu_{f}\left(s,t\right)=\sum_{i}\mu_{i}\left(s,t\right)w_{i}$$

where $\mu_{f}\left(s,t\right)$ is determined by individual predictions, $\mu_{i}\left(s,t\right)$, from the GAMs generated by bootstrap sampling and their weights, $w_{i}$that is proportion to the square of R^2^:

(4) wi=Ri22∑iRI22

Further, the predictions outputted the uncertainty of predictions by calculating the standard deviation of all the predictions from all the models:

(5) $$\sigma_{f}\left(s,t\right)=\sqrt{\frac{\sum_{I}w_{i}{\left(\mu_{i}\left(s,t\right)-\mu_{f}\left(s,t\right)\right)}^{2}}{\frac{M-1}{M}\sum_{i}w_{i}}}$$

where $\sigma_{f}\left(s,t\right)$ is the standard deviation from the output of multiple models, and *M* is the number of nonzero weights.

#### 2.4.4. Kriging of Spatiotemporal Residuals

The daily residuals were modeled for their spatiotemporal patterns. A reasonable assumption is the stable spatial domain after removal of local means [59]. Based on this assumption, the residuals after removal of the means present spatial pattern (autocorrelation) and the variogram could be used to capture such autocorrelation. Consequently, based on the variogram fit, we can infer the residuals for any location under the study domain. Kriging is a classical approach and the readers can refer to [60] for details. Here, based on the stable domain theory after removal of local means [49], kriging was used to estimate the residuals:

(6) $$\varepsilon \left(s_{0},t\right)=\sum_{i=1}^{N}\lambda_{i}\varepsilon \left(s,t\right)$$

where $\varepsilon \left(s_{0},t\right)$ is the residual for a target location to be estimated, $s_{0}$ with time point, t, ε(s,t) is the residual from the estimated mean of the training sample at the location $s_{i}$ and the same time point *t*, from the ensemble models. $\lambda_{i\;}$is the weight for $\varepsilon \left(s_{i},t\right)$ determined by the fitted variogram.

Due to the complexity and instability of directly fitting the spatiotemporal variogram, we simplified variogram fitting by independently fitting daily residuals. Thereby, we can avoid the complex assumption of temporal stability besides spatial stability for directly fitting and improved the effectiveness of parameter estimates for the variogram.

By leave-one-out-cross-validation, we selected the exponential model from multiple models (spherical, circular, exponential, Gaussian, linear models) [61] to simulate the variogram with their optimal performance.

(7) $$\gamma \left(d,t\right)=\{\begin{array}{c} c_{0}\left(t\right)+c\left(t\right)\left(1-exp\left(-\frac{d}{a_{0}\left(t\right)}\right)\right)\\ 0,\;\;\;h=0 \end{array},\;h\;>\;0$$

where γ(d,t) is the variogram function for the residuals, ε(s,t) to be fit, *d* is spatial distance, *t* is the temporal index (day index), $c_{0}\left(t\right)$ is nugget, c(t) is the sill and $a_{0}\;$is the parameter to be estimated.

For variogram fitting, three important parameters—range, nugget and sill—were estimated. Nugget reflects the variance at the discontinuity at the origin, sill is the limit of the variogram tending to infinity lag distances, and range is the distance in which the difference of the variogram from the sill becomes negligible [58]. In our model, the variogram was used to derive the covariance between different locations at the same time that was used in the weighted regression in Equation (6).

Using the daily residual variogram models, we simulated temporal changes in the parameters (range, partial sill, and nugget) for the residual variogram models for 2014. For the predictions, we created grid interpolations for daily residuals for a year across the study region and then added the corresponding estimate of the residual to the bagging prediction estimate to arrive at our final predictions. Therefore, we improved the final predictions by incorporating non-linear associations and residual spatial autocorrelations. The variogram and prediction residuals were completed in ArcGIS (version 10.3, ESRI, Sacramento, CA, USA) using the geostatistical analyst module [62], and the nugget and sill variogram parameters were optimized through cross validations with a focus on the estimation of range parameters.

#### 2.4.5. Validation and Independent Test

To examine the predictive performance of our models under different scenarios, we designed six models with different predictor combinations: Model 1, GAM with no use of the PM_10_ predictor and residual kriging; Model 2, GAM with the PM_10_ predictor but without residual kriging; Model 3, bagging with no use of the PM_10_ predictor and residual kriging; Model 4, bagging with no use of the PM_10_ predictor but with residual kriging; Model 5, bagging with the PM_10_ predictor but without residual kriging; and Model 6, bagging with the PM_10_ predictor and residual kriging. In the six models, the PM_10_ and PM_2.5_ variables were log transformed and transferred back by the exponential function. Bagging predictions for the training dataset were similar to the 63.2–36.8% cross validation. We also conducted a 10 × 10-fold cross-validation for Models 1 and 2. The R^2^ value, root-mean-square error (RMSE), and residual plots between the predicted and observed values were used to compare the performances of the different models.

Independent test was also conducted using Model 4 trained by the 2014 data to predict the 2016 monthly mean PM_2.5_ levels of the 30 sub-regions (Figure S2 of Supplemental Materials) of Shandong Province. Due to the missing PM_2.5_ monitoring station based data for 2016, we just compared the predicted monthly sub-regional mean level of PM_2.5_ with the measured PM_2.5_ level derived from the Ministry of Environmental Protection of People’s Republic of China to illustrate our method’s applicability. Monthly averages of PM_2.5_ were summarized over daily predicted or measured values. Block kriging was used to derive the regional means based on the point estimated or measured values.

## 3. Results

### 3.1. Data Summary

**Figure 4:**
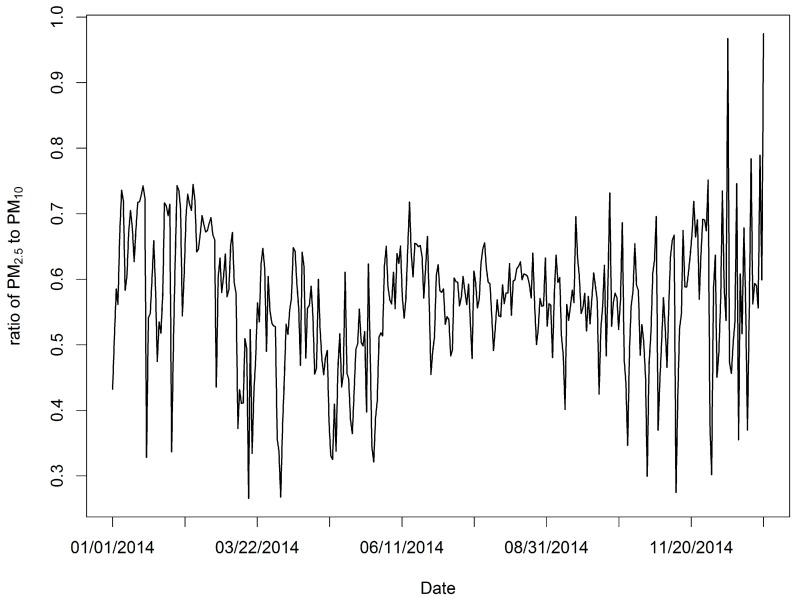
Daily averages of the ratio of PM_2.5_ to PM_10_ over all the monitoring stations across 2014.

We obtained 35,040 measurements for all 96 sample locations, with a rate of missing daily values of 4%, and the SVD method was used to complete the training sample. The daily mean PM_2.5_ and PM_10_ values in the study domain are shown in Figure 2. For all measurements, the mean PM_2.5_ concentration was 73.86 μg/m^3^, while the mean PM_10_ was 128.80 μg/m^3^. The summer and autumn concentrations were obviously lower than the spring and winter concentrations (Figure 2). The PM_2.5_ across the 96 stations varied considerably from approximately 1 μg/m^3^ to nearly 600 μg/m^3^ (standard deviation: 50.79 μg/m^3^), whereas the PM_10_ levels across the 96 stations also varied greatly from roughly 3 μg/m^3^ to nearly 690 μg/m^3^ (standard deviation: 77.37 μg/m^3^). The highest PM_2.5_ mean concentration measured for the period was 130.22 μg/m^3^, and a relatively high PM_10_ value (158.55 μg/m^3^) was measured at the 1624A station located in the northwest part of the province (Figure 1). This figure is much higher than the other observed levels. The highest daily PM_2.5_ concentration for the period was 592.33 μg/m^3^ (PM_10_: 658.92 μg/m^3^), and it was recorded at the 1657A station located in the central part of the province on January 19, 2014. We found 50 stations in the study domain with PM_2.5_ annual mean concentrations exceeding 75 μg/m^3^. We also examined the means of the PM_2.5_ to PM_10_ ratios across daily timelines for 2014 (Figure 4), and the ratios appear to follow a dominant trend with minor variations (mean: 0.57 μg/m^3^ with a standard deviation of 0.10 μg/m^3^, with slightly lower levels in summer than winter).

### 3.2. Predictors

Using univariate and multivariate GAMs (generalized additive models), we explored the non-linear associations of each predictor with PM_2.5_ as well as their respective contributions to the variance. Table 1 presents the variance explained by each predictor of the univariate and multivariate GAM models with and without the inclusion of PM_10_. The results showed that the highest contributions were observed with the co-located PM_10_ (accounting for 67.97–73% of the variance) and the second highest contributions were observed with the first temporal basis function (accounting for 4.84–37% of the variance) in the univariate and multivariate models. For the univariate model, the day of the year and humidity levels accounted for the third and fourth highest contributions to PM_2.5_ variability, respectively. In addition to the co-located PM_10_ and the first temporal basis function, the other predictive variables varied in their contributions in terms of the variance between the univariate model and the multivariate model. For example, although the day of the year explained 14.7% of the variance in the univariate model, it only accounted for a small portion (0.73–2.38%) of the variance in the multivariate model. Similarly, the contributions of AOT (aerosol optical thickness), mean specific humidity and the number of emission plants were also lower for the multivariate models than the univariate models (AOT: 7.38% vs. 0.48–4.77%; humidity: 9.08% vs. 0.48%; number of emission plants: 4.48% vs. 0.97–1.67%). Finally, the traffic index (including roadway lengths within a 10 km buffer of a monitoring location and distances to a primary roadway) made various contributions to explaining the variance (2.62% vs. 1.45–2.86%; 1.73% vs. 1.21–2.15%, respectively).

**Figure 5:**
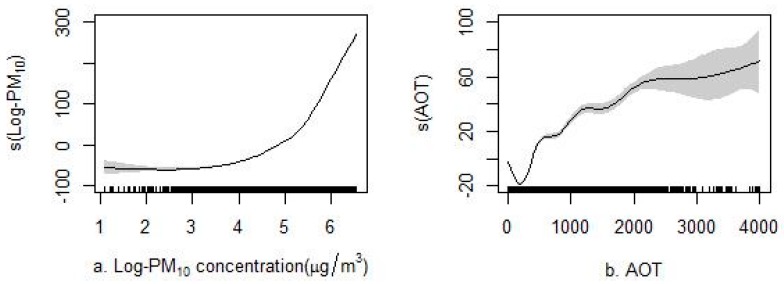

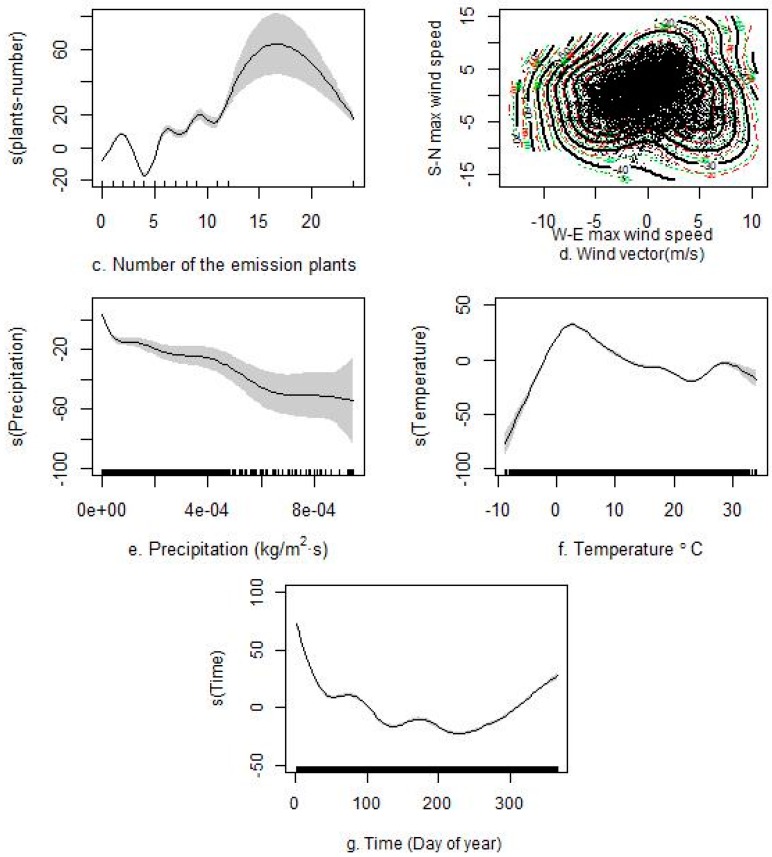
The non-linear associations between each predictor and PM_2.5_. The non-linear association between (**a**) Log-PM_10_ and PM_2.5_; (**b**) aerosol optical thickness (AOT) and PM_2.5_; (**c**) number of the emission plants and PM_2.5_; (**d**) wind and PM_2.5_; (**e**) precipitation and PM_2.5_; (**f**) temperature and PM_2.5_; (**g**) day of year and PM_2.5_.

Figure 5 and Figure S3 of the Supplemental Materials section present the non-linear associations between each predictor and PM_2.5_. The variance explained by the non-linear models was up to 10% than that of the linear models. As shown in Figure 5, the GAMs showed a good ability to capture the non-linear associations between the predictive variables and PM_2.5_. The varying associations (e.g., PM_10_ non-linear associations, including critical varying points, such as 148 μg/m^3^ with PM_2.5_ (also known as 5 log PM_10_) in Figure 5a) captured by the GAMs were critical because they better captured the varying trends between influential factors and concentrations than the linear model. For example, the higher concentrations measured in winter compared with those in summer for the day of the year parameter (Figure 5g) could not be quantified in the linear models. Similarly, varying non-linear associations were also observed for the AOT, the number of emission plants, precipitation, and air temperature.

### 3.3. Comparision of the Different Models and Independent Test

Table 2 shows the results of the six models. Model 1 did not use PM_10_ as a predictor or perform a kriging interpolation of the residuals, and it achieved the worst performance, generating a CV R^2^ of 0.53 and the highest RMSE of 34.69 μg/m^3^. The bagging method (Model 3) results were also similar to that of the ten-fold cross-validation and achieved a similar predictive performance as Model 1; in addition, this method was able to output the standard deviation as a measure of uncertainty. Although the co-located PM_10_ significantly contributed to explaining the variance (28–36%), the kriging interpolation of residuals was able to explain a large proportion (33%) of the variance (Model 4 vs. Model 3) when PM_10_ was not used as a predictor. When PM_10_ was used as a predictor, the kriging interpolation of the daily residuals improved the model’s ability to explain the variance by 7% (Model 6 vs. Model 5). When the PM_10_ predictor was not used, the final model (Model 4) achieved a CV R^2^ of 0.86, and when the PM_10_ predictor was used, the final model (Model 6) achieved a CV R^2^ of 0.89. The plots of predicted vs. measured values and their residual (Models 4 and 6) are shown in Figures S4 and S5 of Supplemental Materials.

Our independent test shows the R^2^ of 0.73 for sub-regional monthly PM_2.5_ averages. Figures S6 of Supplemental Materials shows the scatter plot of predicted vs. measured values and their residual plot.

### 3.4. Variogram Modeling of the Residuals

**Figure 6:**
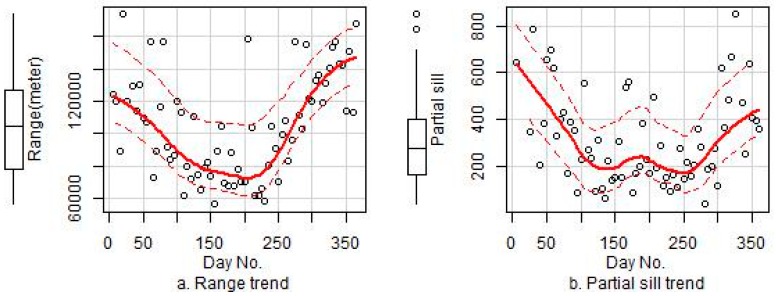

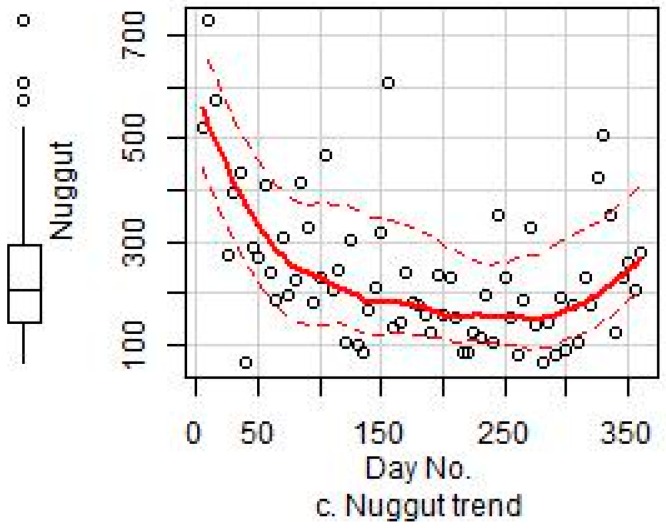
The temporal trends of the three variogram parameters for the residuals of Model 4: (**a**) the temporal trend of range; (**b**) the temporal trend of partial sill; (**c**) the temporal trend of nugget.

As shown in Table 2, the daily residual kriging interpolation considerably improved the predictive performance and accounted for 33% of the explained variance in the model that did not use the PM_10_ predictor. An exponential model was used to fit the variogram of the residuals. Three parameters for the variogram model (range, partial sill, and nugget) were measured over all 365 days of 2014 to explore the temporal patterns of daily residuals. Table 3 shows the variogram parameters for Models 4 and 6, and Figure 6 presents the temporal trends with the fitted curves of the three parameters for 2014. The results show lower range, partial sill, and nugget values in summer than in winter, and this trend may have been caused by the higher concentrations of PM_2.5_, larger influential range, and stronger spatial auto-correlations in winter than summer.

After quantifying the variogram from the exponential model, ordinary kriging was used to interpolate the residuals. The final PM_2.5_ prediction included the output of the bagging approach and the estimate of the specific-time residual. To extend our models to other years, we assumed limited changes in the variograms for PM_2.5_ across different years and overlaid the day-of-year interpolated surfaces of the residual (obtained by the kriging method in ArcGIS) with the target location within the study region to extract the residual estimate for a certain day of the target year.

### 3.5. Uncertainty

**Figure 7:**
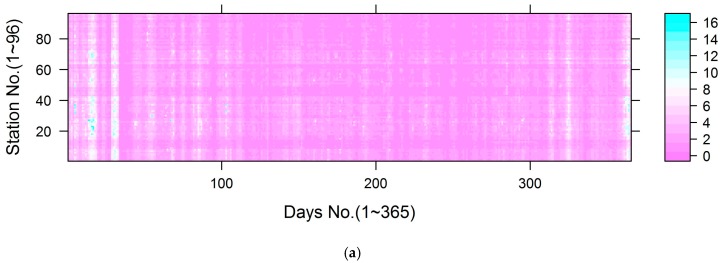

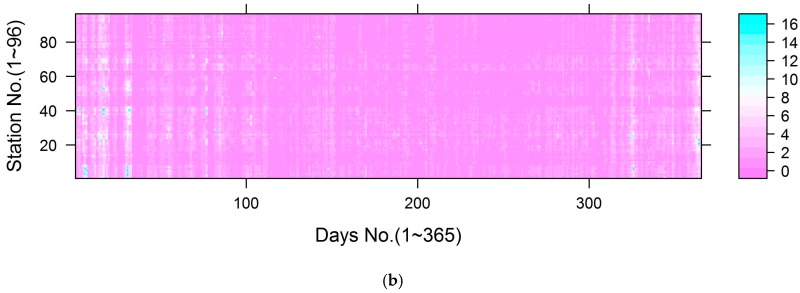
The distribution of standard deviation across monitoring stations: (**a**) the distribution of standard deviation for Models 3 and 4; (**b**) the distribution of standard deviation for Models 5 and 6.

As illustrated, the bagging approach integrated the predictions from 1000 models to generate a stable estimate with standard deviations as a measure of uncertainty. Using the estimated standard deviation, we could also generate 95% confident intervals for the estimate [63,64]. The standard deviation is summarized in Table 4, and the standard deviation distribution is shown in Figure 7. The results show small uncertainty (only 5.2% of the standard deviations exceeded 5 μg/m^3^ for Models 3 and 4, and only 1.9% of the standard deviation exceeded 5 μg/m^3^ for Models 5 and 6).

## 4. Discussion

In this paper, we proposed an ensemble spatiotemporal modeling approach for robustly predicting PM_2.5_ concentrations. For the individual model, we used a GAM (generalized additive models) to determine the non-linear association between PM_2.5_ and multiple predictors (meteorological patterns, traffic indices, season variations, the number of emission sources, and land-use patterns). In particular, we extracted temporal basis functions from the monitoring stations to represent the seasonal PM_2.5_ variability for the study region, which accounted for a large portion of the explained variance. We then used the bagging method to sample the dataset to train 1000 individual models and derived stable ensemble predictions with standard deviations as an uncertainty measure. Then, the kriging method was used to model the residuals from the ensemble predictions to estimate the daily PM_2.5_ residuals throughout a year (365 days) for the study region. For the model that did not include PM_10_ as a predictor, the daily residual kriging achieved a similar predictive performance (R^2^: 0.86 vs. 0.89) as the model using PM_10_ as a predictor. Strong spatial autocorrelations of the residuals accounted for a considerable portion (33%) of the explained variance when co-located PM_10_ values were not included. These results denote the usefulness of residual spatial autocorrelations for predicting PM_2.5_ when PM_10_ measurements are missing as observed for our study location of Shandong Province, China. To our knowledge, previous studies that have performed the same predictions [22,23,65] have only reported R^2^ values of 0.54–0.81, and few studies have achieved a similar estimation accuracy for PM_2.5_ without using PM_10_ as a predictor.

This study also illustrates the important contributions of non-linear associations in the models [19,20,22,23]. The final results show a considerable predictive improvement through the use of non-linear additive models. The results reveal a notable positive non-linear association between PM_2.5_ and PM_10_, AOT (aerosol optical thickness), the number of emission plants, and air temperature, with varying fluctuations found for different predictor intervals (Figure 5a–c,f), whereas a negative association was observed between PM_2.5_ and precipitation (Figure 5e). For the wind vector terms, the PM_2.5_ concentrations tended to decline as the wind speed increased in the south-north and east-west directions because of complex interactions (Figure 5d). Such associations are generally consistent with previous conclusions [29,49].

As a regional pollutant, PM_2.5_ is affected by various factors, including meteorological parameters, emissions sources, and traffic indices [18,19,22,66]. PM_10_ consists of PM_2.5_ and other components, and it is strongly correlated with PM_2.5_; therefore, co-located PM_10_ is a primary predictor of PM_2.5_ levels and explained most of the variance in the multivariate models. Following PM_10_, the first temporal basis function was the second most important predictor. The temporal basis functions captured the seasonal variability of PM_2.5_ levels for the study region. For the model with PM_10_ used as a predictor, the first temporal basis function accounted for roughly 5% of the total variance. However, in the multivariate model (Model 3) without PM_10_, meteorological parameters (including precipitation, wind speed, temperature, and humidity) accounted for only 4.55% of the variance. In addition, traffic indices accounted for 5.01% of the variance; emission plants accounted for roughly 3.34% of the variance; and AOT and NDVI (normalized difference vegetation index) accounted for 4.77% and 0.24% of the variance, respectively. Previous studies of certain regions show weak correlations between AOT and surface PM_2.5_ because the surface reflectance ratio between visible and shortwave infrared channels of the AOT product was underestimated [67]. Our study also illustrates the limited contributions of AOT and NDVI as predictors.

For the models using PM_10_ as a predictor (Models 5 and 6), co-located PM_10_ accounted for most (67.97%) of the variance, whereas the other predictors together accounted for only 13.33%. This finding illustrates that PM_10_ captured a major part of the spatiotemporal variability in PM_2.5_. Without the PM_10_ predictor, the other variables accounted for roughly 53.2% of the variance. In addition to the first temporal basis function (accounting for 26.71%), the other predictors together accounted for only 26.49% of the variance.

Unfortunately, historical PM_2.5_ and PM_10_ measurements are not available for China and many other countries across the globe [16,68]. Even today, the spatial coverage of the PM_2.5_ and PM_10_ monitoring network are limited in China. To comprehensively monitor PM_2.5_ pollution levels and assess their cumulative health effects on humans, reliable estimates of PM_2.5_ concentrations at fine spatiotemporal resolutions should be performed using the limited available PM_2.5_ monitoring data for time periods without available data over extensive spatial areas. Unfortunately, accurately predicting PM_2.5_ levels at high spatiotemporal resolutions is difficult without relevant data on co-located pollutants. In this paper, we explored the use of kriging interpolations of daily residuals, and the results show considerable improvements in predictive performance. Whereas the GAM already took both spatial and temporal information by the covariates, such spatial and spatiotemporal covariates couldn’t fully capture the spatiotemporal variability of PM_2.5_ and so the performance is not so good without use of spatiotemporal residuals or PM_10_. The daily residual’s kriging captured an important portion of PM_2.5_ spatiotemporal variability not captured by GAMs. Although a previous study also employed residual kriging interpolations [69], the variogram modeling of daily residuals remains poorly researched. Therefore, we analyzed the variogram patterns of residuals for a one-year period and performed a cross validation to illustrate the generalizability of the proposed method. As a regional pollutant, PM_2.5_ exhibited stronger effects and spatial autocorrelations than nitrogen oxide. Spatial autocorrelations of the residuals were better able to explain the spatial variability of PM_2.5_ than nitrogen oxide pollutants [49]. Because of the considerable effects of PM_2.5_, particularly for winter in Shandong Province, the residual kriging method was applicable despite the limited number of PM_2.5_ monitoring stations examined and large spatial distances between the monitored samples. As shown in the results (Figure 6), PM_2.5_ in winter showed effects over a longer time period and a greater spatial area than those observed in summer. We expected PM_2.5_ to have an effect over a longer time period (a longer range) and to present higher concentrations (also resulting in higher partial sill and nugget values) in winter than summer. Our variogram modeling of the daily residuals captured these spatial autocorrelation trends to compensate for the gap in the variance explained due to the missing PM_10_ predictor, and thus improved the accuracy of our final predictions. In practice, co-located PM_10_ and other pollutant measurements are not usually available. Thus, Model 4 (using residual kriging interpolations but not PM_10_ as a predictor) has the potential for use in a greater number of applications than the other models that used PM_10_ as a predictor. Our cross-validation results show that Model 4 achieved high levels of accuracy and the results were slightly better than those of Model 5 (using PM_10_ as a predictor but not the residual kriging interpolation) (R^2^: 0.86 vs. 0.82), although the results were slightly less accurate than those of Model 6 (using PM_10_ and residual kriging) (R^2^: 0.86 vs. 0.89). The R^2^ value of Model 6 was only 7% higher than that of Model 5, which may have been related to the spatiotemporal PM_10_ values, which accounted for a major portion of the variance explained in Model 6; whereas the remaining 7% that was not captured by the predictors was explained by the spatial autocorrelations of the residuals.

Based on individual GAMs, our approach introduced the bagging technique to obtain the stable prediction with standard deviation as an uncertainty indicator. Thereby, our prediction could be robust and less over-fitting although our performance is pretty similar to the individual model’s output from the other approaches [13,24,25,65,70]. On the other hand, due to characteristics of strong spatial autocorrelation of PM_2.5_ concentration, our approach could considerably improve performance even without use of the PM_10_ predictor. Thus, for practical prediction, our approach does not need the extraction of complicated covariates such as output of the chemical transport model (GEOS-CHEM) and is less costly with a similar performance at high spatiotemporal resolution. Production of PM_2.5_ is complicated and varies with different regions. While some approaches may have good performances in other regions [13,65,70], they may be not applied to China as our approach, due to unavailability of some predictive covariates or differences in geographical and meteorological factors and emission sources.

This study presents several limitations. First, we extracted the first and second temporal basis functions from regular monitoring data to represent the study region’s seasonal variability in PM_2.5_. This procedure could have resulted in overfitting. To determine the influence on the final results, we conducted a 10 × 10 cross-validation and independent test, and the results (CV R^2^: 0.86–0.89; 0.73 for independent test) show high levels of predictive accuracy, thereby illustrating the limited influence of the temporal basis functions extracted from the predictions. Second, our variogram modeling of the daily residuals was not systematic, which may have affected the applicability of the proposed spatiotemporal approach. Our validation and independent results show that such effects are limited for extensive applications. Third, our spatiotemporal modeling approach was based on monitoring data for Shandong Province, China; thus, its applicability may be limited. PM_2.5_ pollution has a larger sphere of influence in northern regions of China, including Shandong Province, and the spatial autocorrelation of residuals of PM_2.5_ concentrations was markedly strong. Thus, the use of the residual kriging method for less dense monitoring networks could still capture the spatiotemporal variability of PM_2.5_ and account for a large portion of the variance. For regions with lower PM_2.5_ pollution and sparser monitoring networks, the residual kriging method may not be applicable, and spatial effects modeling may represent a more preferable approach. We have explored and reported on such a method in another study [71].

## 5. Conclusions

This paper has proposed an ensemble spatiotemporal modeling approach for improved prediction of PM_2.5_ even with missing co-located pollutants such as PM_10_. Our approach uses a generalized additive model to quantify the non-linear associations between the predictors and PM_2.5_, and ensemble learning was used to obtain stable predictions, with standard deviations used as an uncertainty measure. In addition, the variogram of the daily residuals was modeled to capture day-of-year patterns for the residuals. The results showed the better CV R^2^ of 0.89 (with co-located PM_10_ used as a predictor) and 0.86 (without PM_10_ used as a predictor) than the previous methods. The results demonstrate that our approach can be used to estimate PM_2.5_ exposure at a good accuracy and indicate its potential applicability for estimating PM_2.5_ exposure in Shandong Province or other similar regions of China.

## Figures and Tables

**Table 1:** Variance explained for each predictive variable.

Predictive Variable (Unit)	Variance Explained in the Univariate Model	Variance Explained in the Multivariate Model (without PM_10_)	Variance Explained in the Multivariate Model (Including PM_10_)
PM_10_ (μg/m^3^)	73.00%	-	67.97%
Aerosol optical thickness (AOT)	7.38%	4.77%	0.48%
Normalized difference vegetation index (NDVI)	3.14%	0.24%	0.24%
Precipitation (kg/m^2^s)	1.75%	0.02%	0.02%
Temperature (°C)	1.08%	2.62%	0.48%
Mean specific humidity (kg/kg)	9.08%	0.48%	0.48%
Roadway length within the 10 km buffer of a monitoring station (m)	2.62%	2.86%	1.45%
Shortest distance of roadway to a monitoring station (m)	1.73%	2.15%	1.21%
Wind speed vector	3.76%	1.43%	0.48%
Area proportion of the factories and mines, oil fields and stone-pit land-use	2.29%	2.15%	0.73%
Area proportion of the forest land-use	2.06%	2.62%	0.48%
Number of the emission plants	4.48%	1.67%	0.97%
Shortest distance to the emission plants	1.70%	1.67%	0.24%
The first temporal basis function	37.00%	26.71%	4.84%
The second temporal basis function	5.79%	1.43%	0.48%
Time (day of year)	14.70%	2.38%	0.73%
Total		53.20%	81.30%

**Table 2:** Comparison of multiple models.

Model	Use of Predictive Variables and Residual Kriging	R^2^	CV ^a^ R^2^	CV RMSE ^b^ (μg/m^3^)
Model 1	GAM with no use of PM_10_ data and residual kriging	0.53	0.53	34.69
Model 2	GAM with PM_10_ data but without residual kriging	0.81	0.81	21.87
Model 3	Bagging without PM_10_ data and residual kriging		0.53	34.79
Model 4	Bagging without PM_10_ data but with residual kriging		0.86	18.85
Model 5	Bagging with PM_10_ data but without residual kriging		0.82	21.82
Model 6	Bagging with PM_10_ data and residual kriging		0.89	17.06

^a^ CV: Cross Validation; ^b^ RMSE: Root Mean Squared Error.

**Table 3:** Summary of variogram parameters for Models 4 and 6.

Model	Parameter	Minimum	1st Qu. ^a^	Median	Mean	3rd Qu. ^a^	Maximum
Model 4	Range	5551	63,780	93,050	107,100	137,000	712,900
	Partial sill	1.65	96.18	208	448.7	507	6560
	Nugget	20.74	97.12	159.6	260.4	284.3	3096
Model 6	Range	4250	60,350	95,660	103,100	144,500	475,200
	Partial sill	0.0839	29.7	71.27	144.4	162.3	2866
	Nugget	0.0912	85.33	146.6	228.1	264.5	2650

^a^ Qu.: Quarter.

**Table 4:** Summary of standard deviation.

Model	Minimum	1st Qu. ^a^	Median	Mean	3rd Qu. ^a^	Max.
Models 3 and 4	0.43	1.33	1.80	2.18	2.54	21.43
Models 5 and 6	0.26	0.79	1.19	1.47	1.76	26.81

^a^ Qu.: Quarter.

## Supplementary Materials

The following are available online at www.mdpi.com/1660-4601/14/5/549/s1, Figure S1: Histogram with normal density plot of PM_2.5_ and PM_10_, Figure S2: Thirty sub-regions in Shandong province, Figure S3: Associations of predictive covariates and PM_2.5_, Figure S4: Residual plots for the measured and fitted PM_2.5_ in Model 4 (no PM_10_ used but including estimates of the residuals), Figure S5: Residual plots for the measured and predicted PM_2.5_ in Model 6 (PM_10_ used and incorporation of estimates of the residuals), Figure S6: Residual plots for the measured and predicted monthly-mean PM_2.5_ in regions, Table S1: An annex table for technical terms.
