# Supplementary material for: An Ensemble Spatiotemporal Model for Predicting PM2.5 Concentrations
Source: Int J Environ Res Public Health. 2017 May 22;14(5):549. doi: 10.3390/ijerph14050549 (PMC5451999; doi:10.3390/ijerph14050549)
Supplement: Supplementary file 1 [file ijerph-14-00549-s001.pdf]

Table S1. An annex table for technical terms.

Name	Description
AOT	Aerosol optical thickness: the degree to which aerosols prevent the transmission of light by absorption or scattering of light; usually extracted from MODIS satellite images.
bagging	A machine learning method for generating multiple versions of a predictor and using these to get an aggregated predictor.
CV R2	Cross validation R-squared
GAM	Generalized additive model
LUR	Land use regression
Residual	Difference between the observed values and the means predicted by individual or multiple GAMs
	Variogram parameters
nugget	At an infinitely small separation distance, the semivariogram often exhibits a nugget effect, which is a value greater than 0 that reflects the variance at the discontinuity at the origin
partial sill	A partial sill is the sill minus the nuggets, and the sill is the limit value at which the semivariogram model attains the range
range	The distance where the semivariogram model first flattens

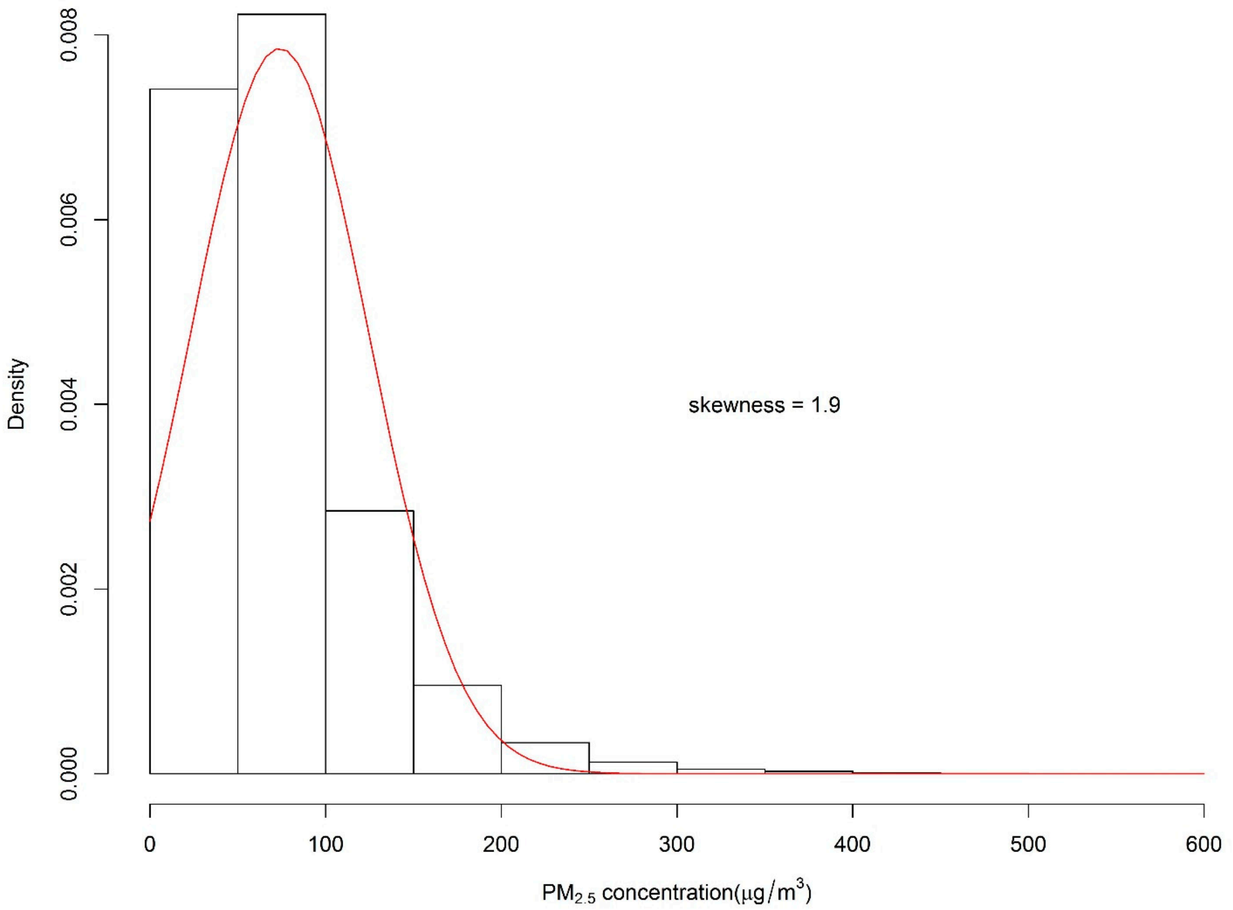

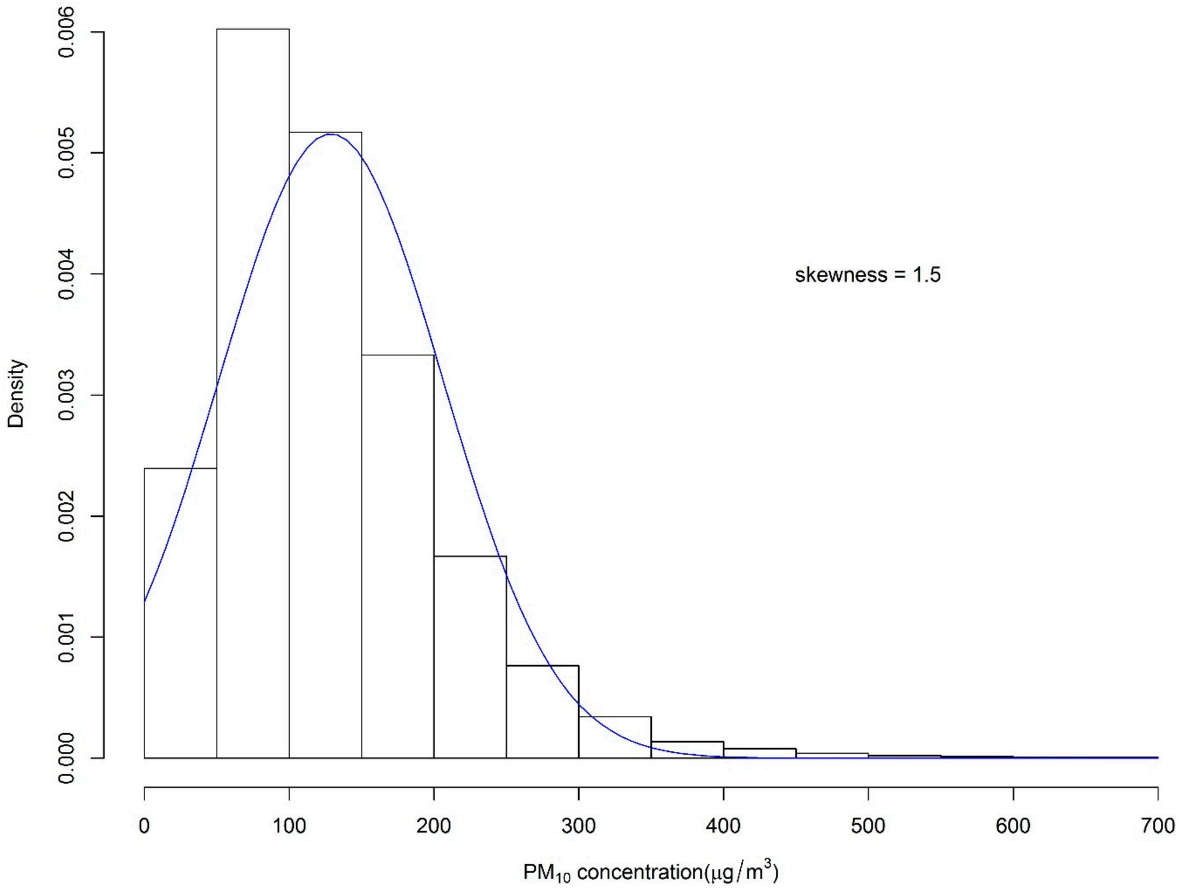

Figure S1. Histogram with normal density plot of $PM_{2.5}$ and PM_{10}.

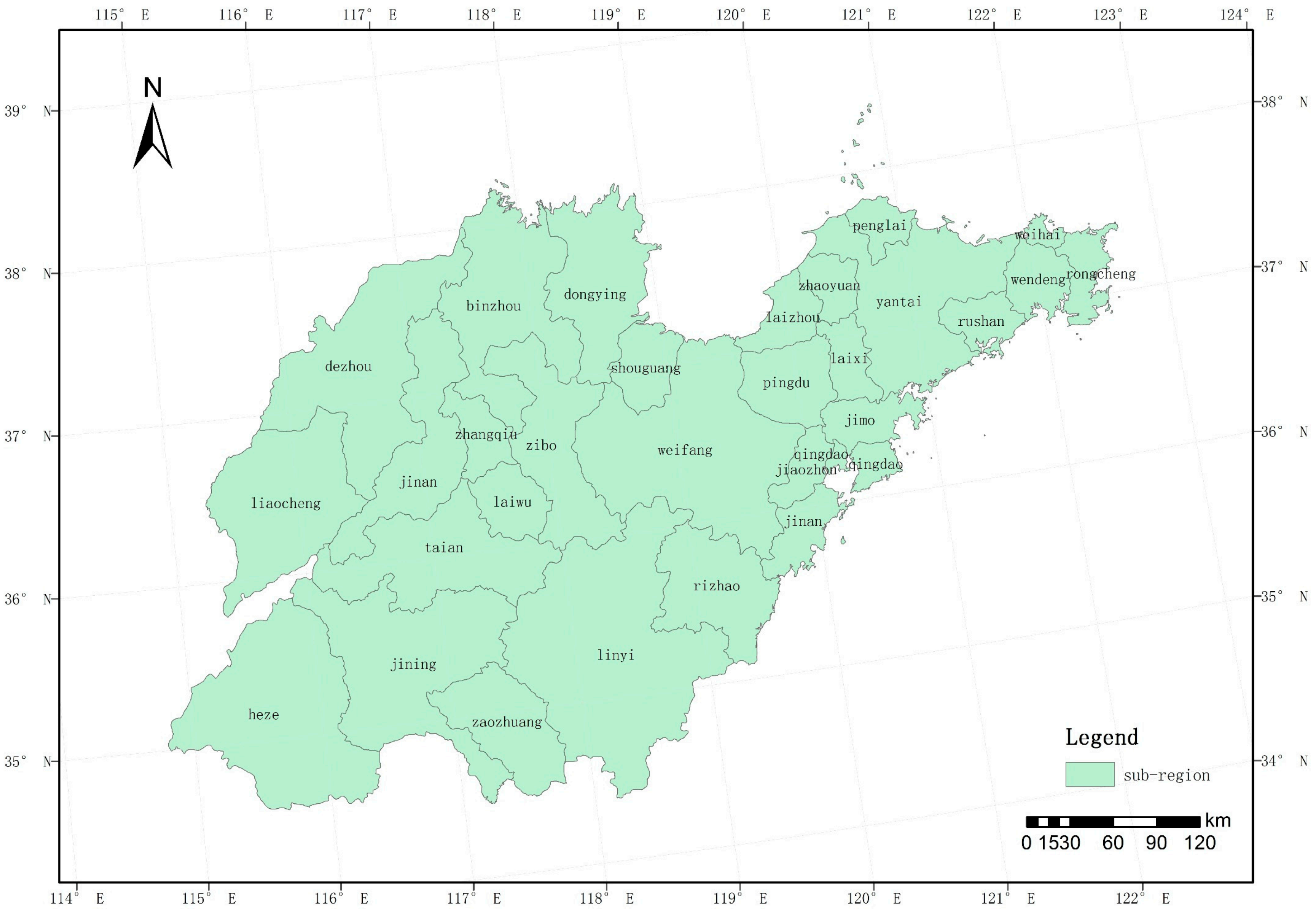

Figure S2. Thirty sub-regions in Shandong province.

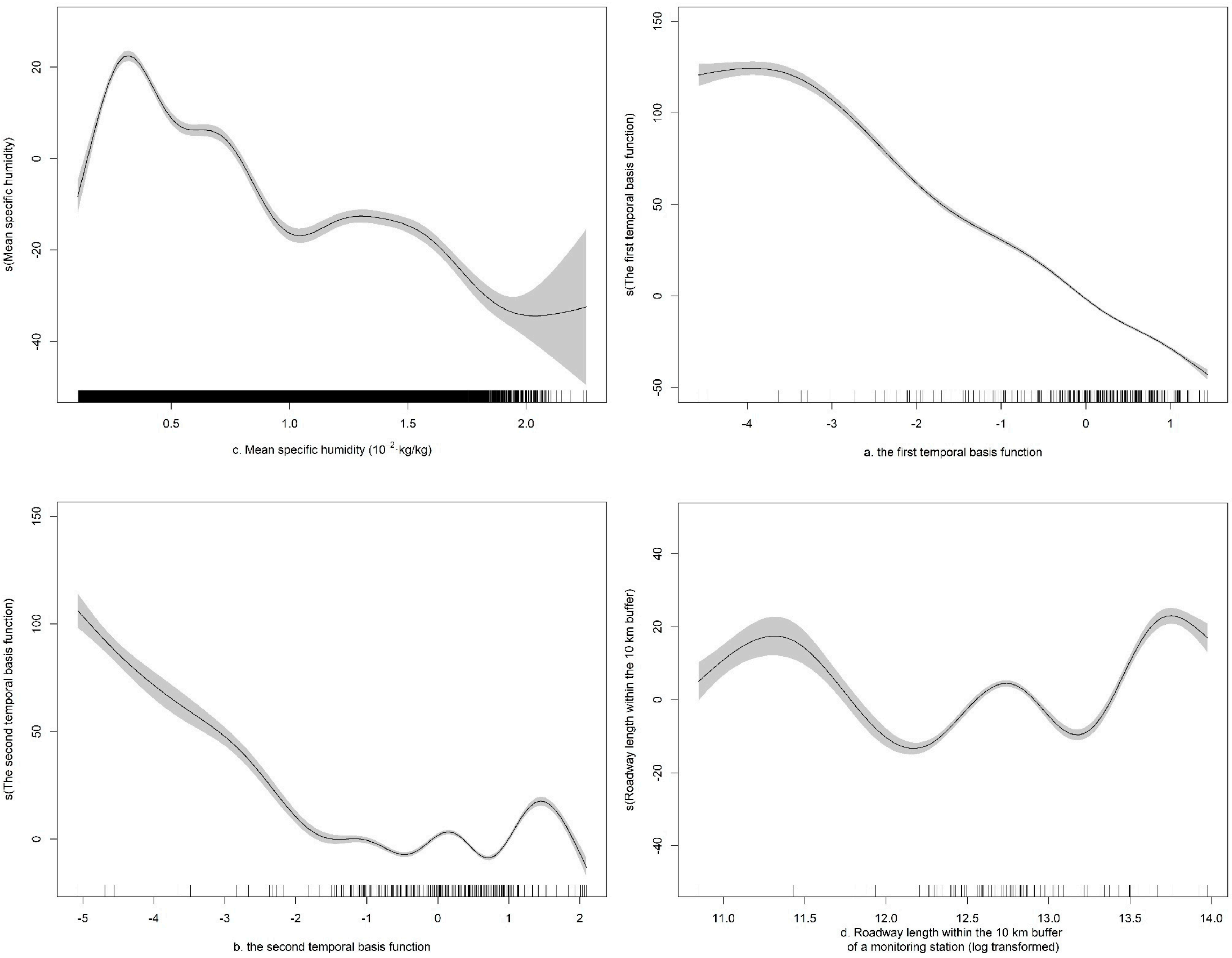
s(Mean specific humidity)
20
0
-20
-40
0.5
1.0
1.5
2.0
c. Mean specific humidity (10 2·kg/kg)
s(The first temporal basis function)
150
100
50
0
-50
-4
-3
-2
-1
0
1
a. the first temporal basis function
s(The second temporal basis function)
150
100
50
0
-5
-4
-3
-2
-1
0
1
2
b. the second temporal basis function
s(Roadway length within the 10 km buffer)
40
20
0
-20
-40
11.0
11.5
12.0
12.5
13.0
13.5
14.0
d. Roadway length within the 10 km buffer
of a monitoring station (log transformed)

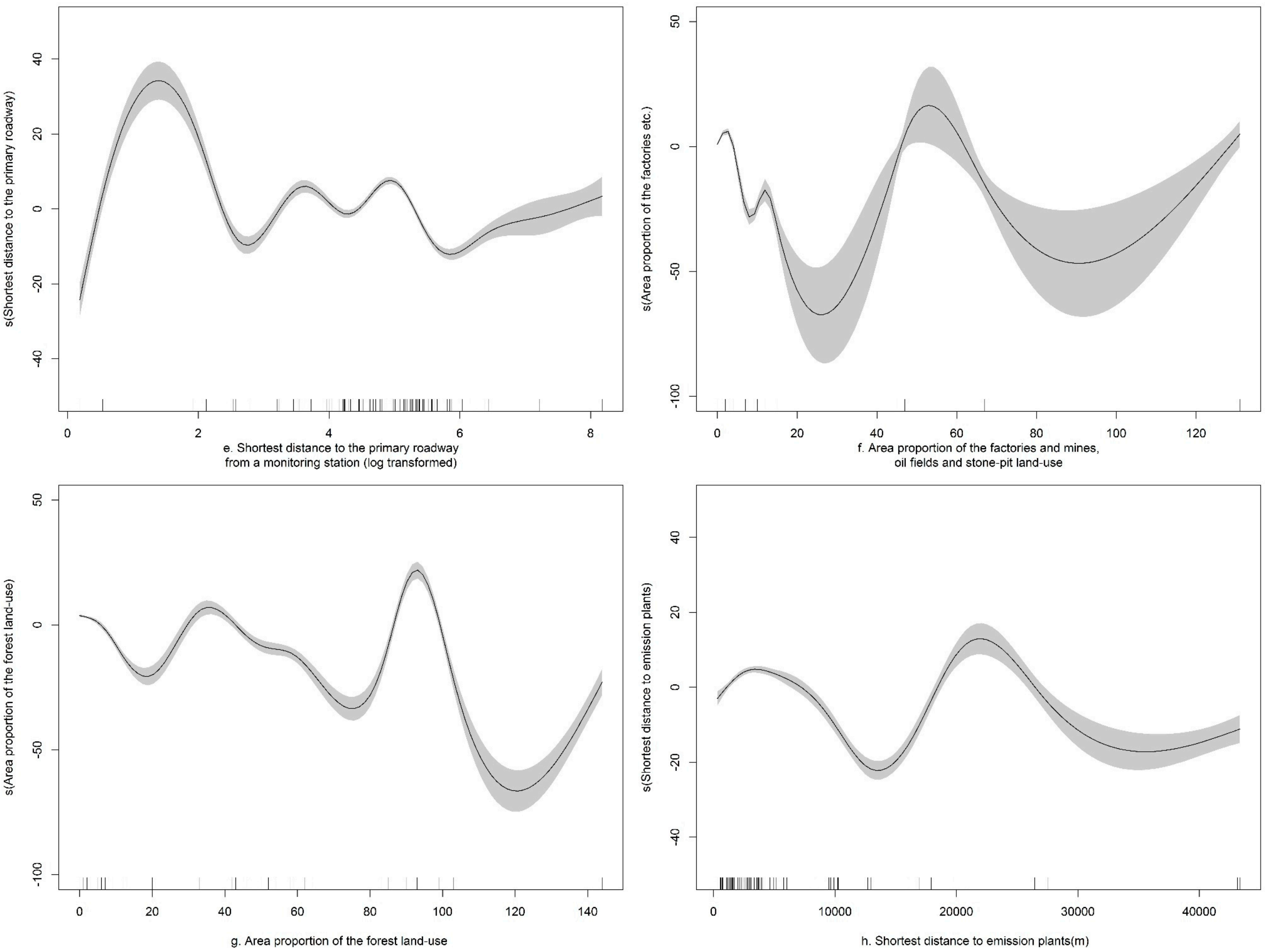

Figure S3. Associations of predictive covariates and $PM_{2.5}$.

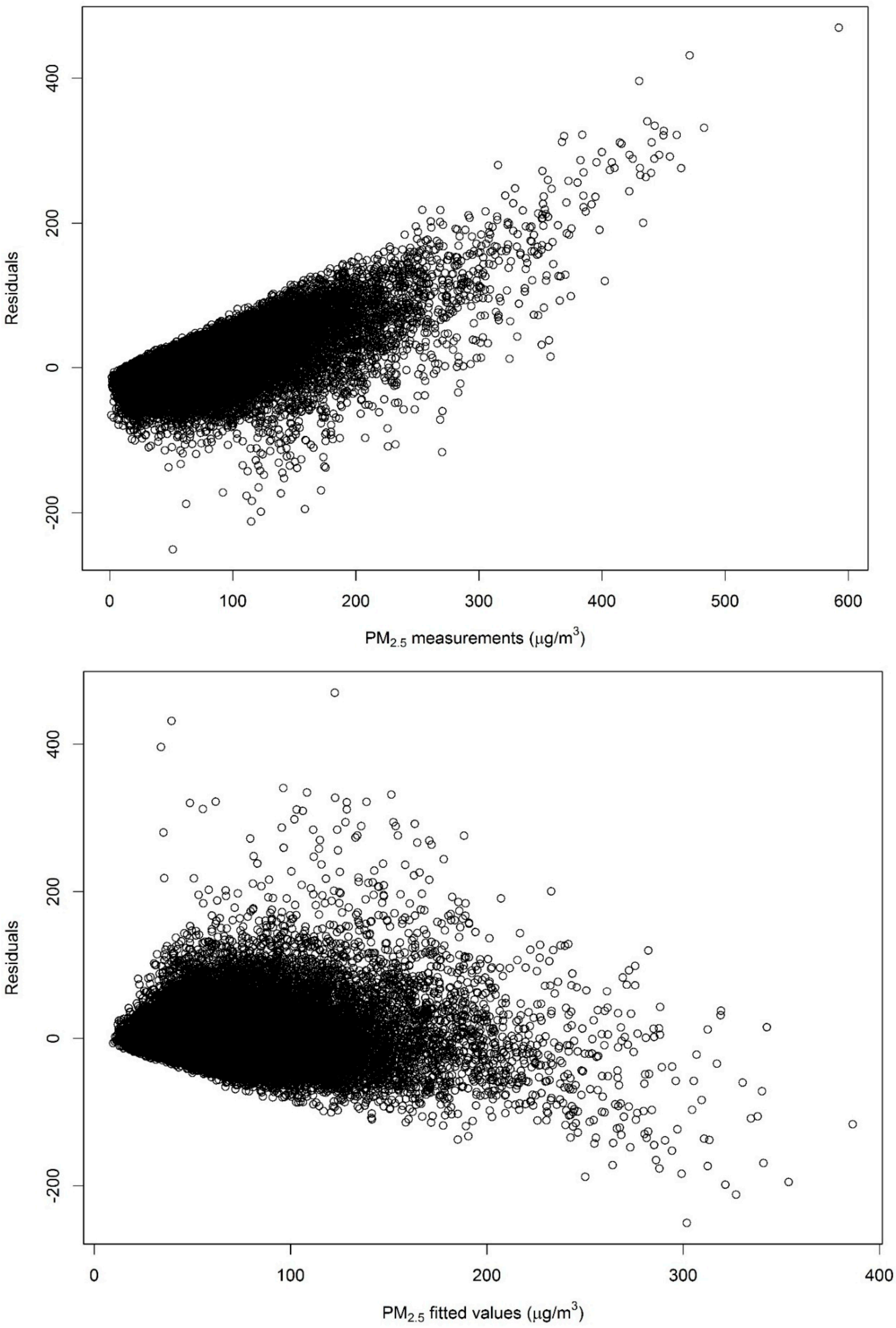

Figure S4. Residual plots for the measured and fitted $PM_{2.5}$ in Model 4 (no PM_{10} used but including estimates of the residuals).

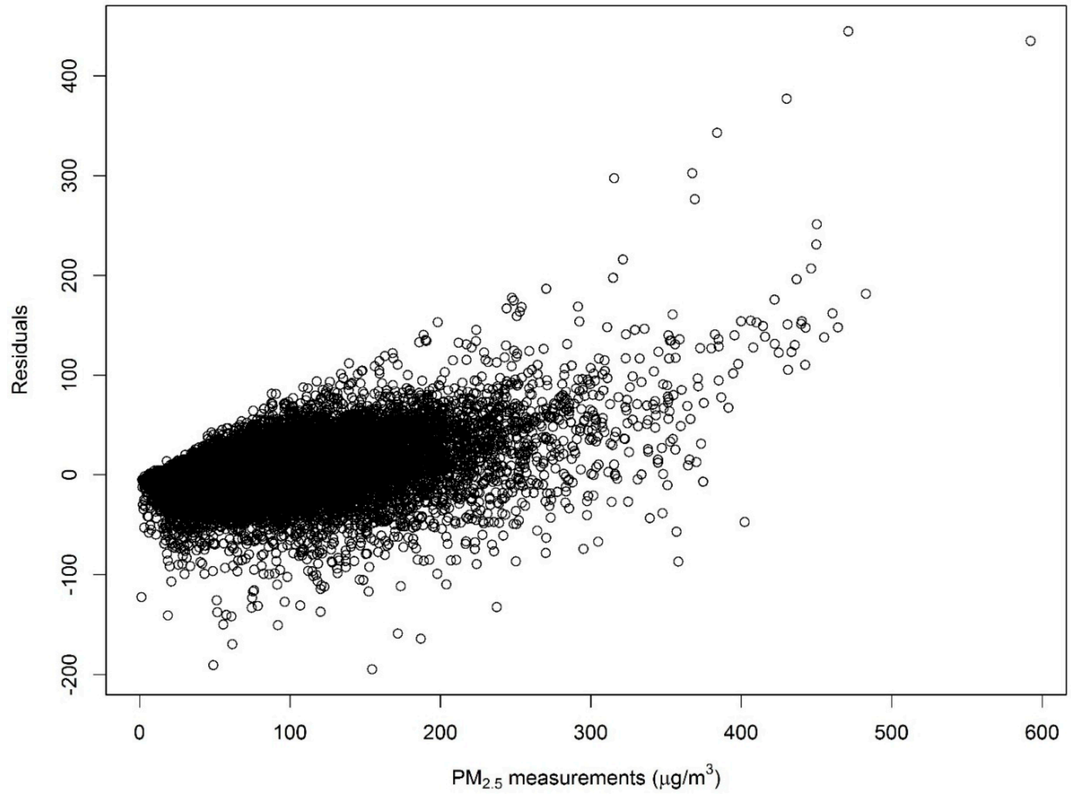

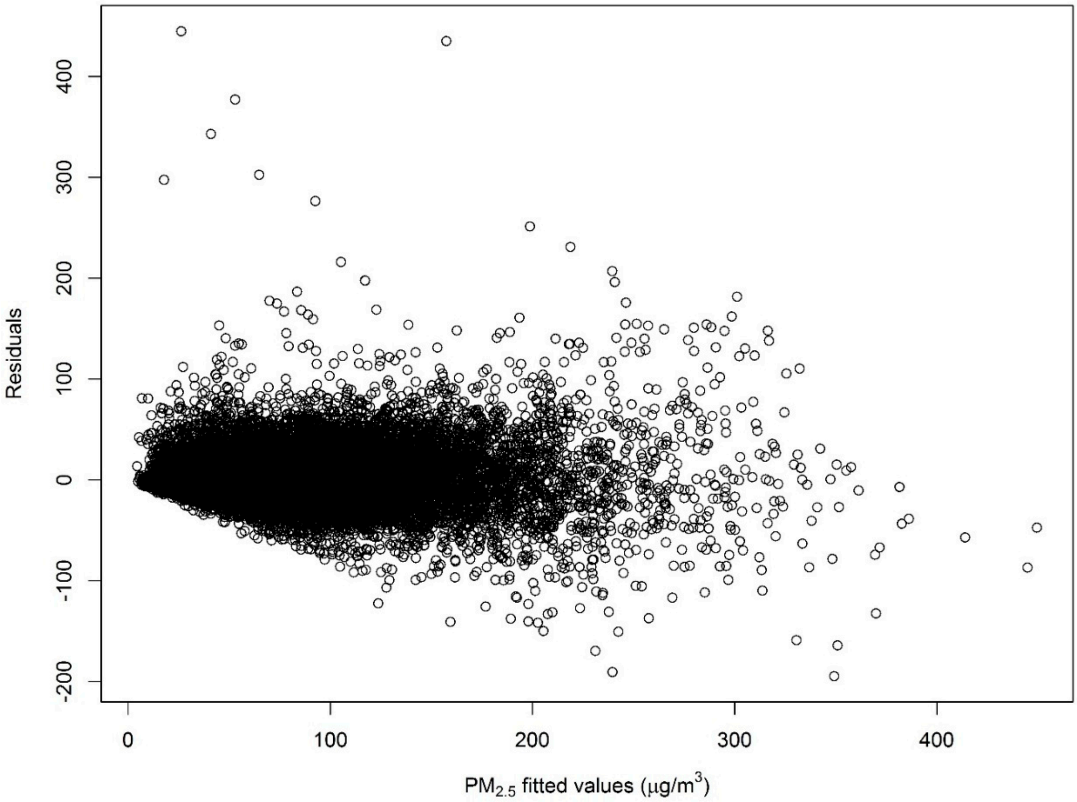

Figure S5. Residual plots for the measured and predicted $PM_{2.5}$ in Model 6 (PM_{10} used and incorporation of estimates of the residuals).

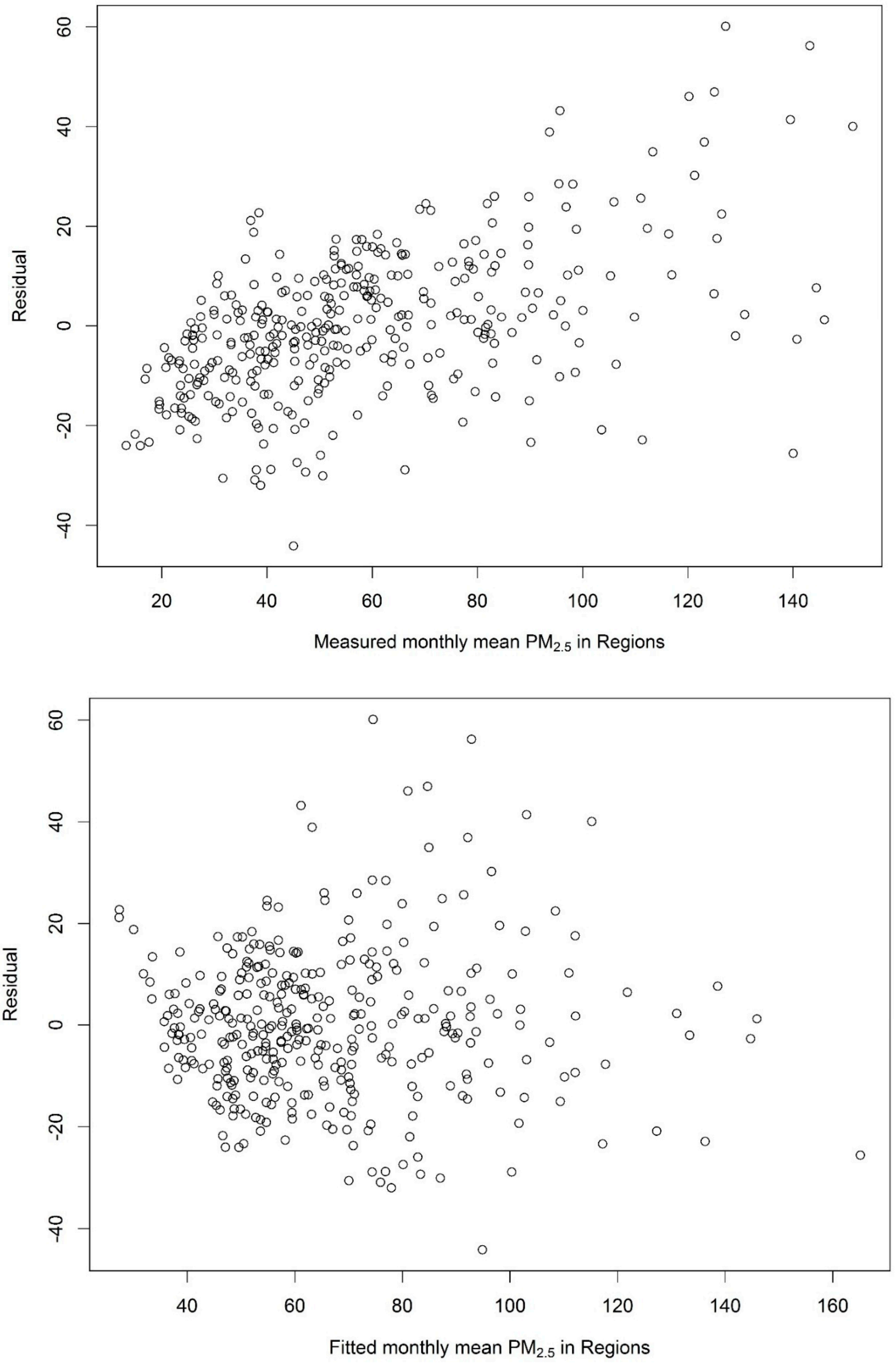

Figure S6. Residual plots for the measured and predicted monthly-mean $PM_{2.5}$ in regions.
